# Supplementary material for: A Systematic Review of Internet of Things in Clinical Laboratories: Opportunities, Advantages, and Challenges
Source: Sensors (Basel). 2022 Oct 21;22(20):8051. doi: 10.3390/s22208051 (PMC9611742; doi:10.3390/s22208051)
Supplement: Supplementary file 1 [file sensors-22-08051-s001.zip › sensors-1951960-supplementary.pdf]

Supplementary Materials

Full-Text Screening (Excluded Studies) (N=23):

Papers that were not concept papers or primary research articles, and that did not belong to any of the focal points of IoT in laboratory processes illustrated in Figure 2 were excluded. Books, conference papers, symposiums commentaries, and review articles were excluded. Non-English studies were omitted as well. Their bibliographies were screened to identify any relevant paper pertaining to our theme.

Did not belong to any focal points of IoT laboratory processes:

1. Chao Y, Jiang W, Wang X, Wang X, Song J, Chen C, Zhou J, Huang Q, Hu J, Song Y. Discovery of efficacy biomarkers for non-small cell lung cancer with first- line anti-PD-1 immunotherapy by data-independent acquisition mass spectrometry. Clin Exp Immunol. 2022; 208(1):60–71. doi: 10.1093/cei/uxac021.
2. Li H, Wang Y, Jiang J, Zhao H, Feng X, Zhao B, Wang L. A Novel Human Microbe-Disease Association Prediction Method Based on the Bidirectional Weighted Network. Front Microbiol. 2019; 10:676. doi: 10.3389/fmicb.2019.00676
3. Lin D, Wang Y, Wang T, Zhu Y, Lin X, Lin Y, et al. Metabolite profiling of human blood by surface-enhanced Raman spectroscopy for surgery assessment and tumor screening in breast cancer. Anal Bioanal Chem. 2020; 412(7):1611-1618. doi: 10.1007/s00216-020-02391-4.
4. Lin D, Wu Q, Qiu S, Chen G, Feng S, Chen R, et al. Label-free liquid biopsy based on blood circulating DNA detection using SERS-based nanotechnology for nasopharyngeal cancer screening. Nanomedicine. 2019; 22:102100. doi: 10.1016/j.nano.2019.102100.
5. Lin X, Wang Y, Wang L, Lu Y, Li J, Lu D, et al. Interference-free and high precision biosensor based on surface enhanced Raman spectroscopy integrated with surface molecularly imprinted polymer technology for tumor biomarker detection in human blood. Biosens Bioelectron. 2019; 143:111599. doi: 10.1016/j.bios.2019.111599
6. Tong T, Gao Q, Guerrero R, Ledig C, Chen L, Rueckert D, Initiative ADN. A Novel Grading Biomarker for the Prediction of Conversion From Mild Cognitive Impairment to Alzheimer's Disease. IEEE Trans Biomed Eng. 2017;64(1):155-165. doi: 10.1109/TBME.2016.2549363.
7. Uehara F, Hori K, Hasegawa Y, Yoshimura S, Hori S, Kitamura M, et al. Impact of Masticatory Behaviors Measured With Wearable Device on Metabolic Syndrome: Cross-sectional Study. JMIR Mhealth Uhealth. 2022 Mar 24;10(3):e30789. doi: 10.2196/30789. PMID: 35184033; PMCID: PMC8990367.
8. Vashistha R, Dangi AK, Kumar A, Chhabra D, Shukla P. Futuristic biosensors for cardiac health care: an artificial intelligence approach. 3 Biotech. 2018;8(8):358. doi: 10.1007/s13205-018-1368-y.
9. Wu C, Zhong J, Lin L, Chen Y, Xue Y, Shi P. Segmentation of HE-stained meningioma pathological images based on pseudo-labels. PLoS One. 2022; 17(2):e0263006. doi: 10.1371/journal.pone.0263006.

10. Xu Y, Wang Y, Lin H, Liu X, Zheng Z, Wang T, Feng S. Serum analysis method combining cellulose acetate membrane purification with surface-enhanced Raman spectroscopy for non-invasive HBV screening. IET Nanobiotechnol. 2020 Feb 1;141:98-104.

Non-English studies:

11. Díaz de León-Castañeda C. Salud electrónica (e-Salud): un marco conceptual de implementación en servicios de salud. Gac Med Mex. 2019;155(2):176-183. doi: 10.24875/GMM.18003788.
12. He Y, Yang B, Xiong S, Li Q. Design of Portable Spirometer Based on Internet of Things of Medicine. Zhongguo Yi Liao Qi Xie Za Zhi. 2018;42(2):103-106. doi: 10.3969/j.issn.1671-7104.2018.02.007.

Books/Conference Papers/Symposiums/Commentaries/Reviews:

13. Azrour M, Irshad A, Chaganti R. IoT and Smart Devices for Sustainable Environment.
14. Calvillo-Arbizu J, Román-Martínez I, Reina-Tosina J. Internet of things in health: Requirements, issues, and gaps. Computer Methods and Programs in Biomedicine. 2021; 208:106231.
15. Dong T, Santos S, Yang Z, Yang S, Kirkhus NE. Sputum and salivary protein biomarkers and point-of-care biosensors for the management of COPD. Analyst. 2020;145(5):1583-604.
16. Gupta N, Paiva S, editors. IoT and ICT for Healthcare Applications. Springer International Publishing; 2020 Aug 12.
17. Inácio PR, Duarte A, Fazendeiro P, Pombo N, editors. 5th EAI International Conference on IoT Technologies for HealthCare. Cham: Springer; 2020.
18. Li Y, Lu H, editors. 3rd EAI International Conference on Robotic Sensor Networks. Springer; 2020.
19. M. S. Packianather, N. L. Munizaga, S. Zouwail and M. Saunders, "Development of soft computing tools and IoT for improving the performance assessment of analysers in a clinical laboratory," *2019 14th Annual Conference System of Systems Engineering (SoSE)*, 2019, pp. 158-163, doi: 10.1109/SYSOSE.2019.8753830.
20. Velliangiri S, Gunasekaran M, Karthikeyan P. Secure Communication for 5G and IoT Networks. Springer International Publishing AG; 2021.
21. Yang X, Wang X, Li X, Gu D, Liang C, Li K, Zhang G, Zhong J. Exploring emerging IoT technologies in smart health research: a knowledge graph analysis. BMC Medical Informatics and Decision Making. 2020;20(1):1-2.
22. Yetisen AK, Martinez-Hurtado JL, Ünal B, Khademhosseini A, Butt H. Wearables in Medicine. Adv Mater. 2018;30(33):e1706910. doi: 10.1002/adma.201706910.
23. Yoo J, Soh JY, Lee WH, Chang DK, Lee SU, Cha WC. Experience of Emergency Department Patients With Using the Talking Pole Device: Prospective Interventional Descriptive Study. JMIR Mhealth Uhealth. 2018;6(11):e191. doi: 10.2196/mhealth.9676.

Supplementary Table S1: Findings for the JBI Critical Appraisal Tool.

Study and Year	1. Is the source of the text clearly identified?	2. Does the source of text have standing in the field of expertise?	3. Are the interests of the relevant population the central focus of the opinion?	4. Is the stated position the result of an analytical process, and is there logic in the opinion expressed?	5. Is there reference to the extant literature?	6. Is any incongruence with the literature/sources logically defended?
Alam, 2015	Yes	Yes	No	Yes	Yes	Unclear
Alonso, 2020	Yes	Yes	No	Yes	Yes	Yes
Austerjost, 2018	Yes	Yes	Yes	Yes	Yes	No
Bibi, 2020	Yes	Unclear	Yes	Yes	Yes	Yes
Celesti, 2020	Yes	Yes	Yes	Yes	Unclear	Unclear
Galindo-Romera, 2017	Yes	Unclear	Yes	Yes	Yes	Yes
Guo, 2021	Yes	Unclear	Unclear	Yes	Yes	Yes
Kalasin, 2020 (1)	Yes	No	Yes	Unclear	No	Yes
Kalasin, 2020 (2)	Yes	Yes	Yes	Yes	Unclear	Yes
Kang, 2018	Yes	Yes	Yes	Yes	Yes	Yes
Ma, 2020	Yes	Yes	Yes	Yes	Yes	Yes
Majdoubi, 2021	Yes	Yes	Yes	Yes	Yes	Yes
Neil, 2018	Yes	Yes	Unclear	Yes	No	Yes
Ochôa, 2019	Yes	Yes	Yes	Yes	Yes	Yes
Porr, 2020	Yes	Yes	Yes	Yes	Yes	Yes
Shumate, 2018	Yes	Yes	Yes	Yes	Yes	Yes
Wang, 2018	Yes	Yes	Yes	No	Yes	Yes
Zhu, 2020	Yes	Yes	Yes	Unclear	Yes	Yes

Checklist includes: Yes, No, Unclear, Not Applicable
